# Analysis of false reasons based on the artificial intelligence RRCART model to identify frozen sections of lymph nodes in breast cancer

**DOI:** 10.1186/s13000-023-01432-7

**Published:** 2024-01-22

**Authors:** Zuxuan Zhao, Cancan Chen, Hanwen Guan, Lei Guo, Wanxin Tian, Xiaoqi Liu, Huijuan Zhang, Jiangtao Li, Tinglin Qiu, Jun Du, Qiang Guo, Fenglong Sun, Shan Zheng, Jianhui Ma

**Affiliations:** 1 Department of Pathology, National Cancer Center/National Clinical Research Center for Cancer/Cancer Hospital, Chinses Academy of Medical Sciences and Peking Union Medical College, Beijing, 100021 China; 2 Digital Health China Technologies Corporation Limited, Beijing, 100080 China; 3 Infervision Medical Technology Co., Ltd, Beijing, 100025 China; 4 School of Health Management, Harbin Medical University, Harbin, 150081 Heilongjiang Province China; 5 Department of Medical Affairs, National Cancer Center/National Clinical Research Center for Cancer/Cancer Hospital, Chinese Academy of Medical Sciences and Peking Union Medical College, Beijing, 100021 China; 6 Department of Pathology, National Cancer Center/National Clinical Research Center for Cancer/Cancer Hospital/ Shenzhen Hospital, Chinese Academy of Medical Sciences and Peking Union Medical College, Shenzhen, 528116 China; 7 Administration Office, National Cancer Center/National Clinical Research Center for Cancer/Cancer Hospital, Chinese Academy of Medical Sciences and Peking Union Medical College, Beijing, 100021 China; 8 Department of Big Data, National Cancer Center/National Clinical Research Center for Cancer/Cancer Hospital, Chinese Academy of Medical Sciences and Peking Union Medical College, Beijing, 100021 China; 9 Healthcare IT Department, Genertec Universal Medical Group Co., Ltd, Beijing, 100062 China; 10 Department of Urology, National Cancer Center/National Clinical Research Center for Cancer/Cancer Hospital, Chinese Academy of Medical Sciences and Peking Union Medical College, Beijing, 100021 China

**Keywords:** Breast cancer, Sentinel lymph node, Artificial intelligence, Frozen section, False reason

## Abstract

**Background:**

Breast cancer is the most common malignant tumor in the world. Intraoperative frozen section of sentinel lymph nodes is an important basis for determining whether axillary lymph node dissection is required for breast cancer surgery. We propose an RRCART model based on a deep-learning network to identify metastases in 2362 frozen sections and count the wrongly identified sections and the associated reasons. The purpose is to summarize the factors that affect the accuracy of the artificial intelligence model and propose corresponding solutions.

**Methods:**

We took the pathological diagnosis of senior pathologists as the gold standard and identified errors. The pathologists and artificial intelligence engineers jointly read the images and heatmaps to determine the locations of the identified errors on sections, and the pathologists found the reasons (false reasons) for the errors. Through NVivo 12 Plus, qualitative analysis of word frequency analysis and nodal analysis was performed on the error reasons, and the top-down error reason framework of “artificial intelligence RRCART model to identify frozen sections of breast cancer lymph nodes” was constructed based on the importance of false reasons.

**Results:**

There were 101 incorrectly identified sections in 2362 slides, including 42 false negatives and 59 false positives. Through NVivo 12 Plus software, the error causes were node-coded, and finally, 2 parent nodes (high-frequency error, low-frequency error) and 5 child nodes (section quality, normal lymph node structure, secondary reaction of lymph nodes, micrometastasis, and special growth pattern of tumor) were obtained; among them, the error of highest frequency was that caused by normal lymph node structure, with a total of 45 cases (44.55%), followed by micrometastasis, which occurred in 30 cases (29.70%).

**Conclusions:**

The causes of identification errors in examination of sentinel lymph node frozen sections by artificial intelligence are, in descending order of influence, normal lymph node structure, micrometastases, section quality, special tumor growth patterns and secondary lymph node reactions. In this study, by constructing an artificial intelligence model to identify the error causes of frozen sections of lymph nodes in breast cancer and by analyzing the model in detail, we found that poor quality of slices was the preproblem of many identification errors, which can lead to other errors, such as unclear recognition of lymph node structure by computer. Therefore, we believe that the process of artificial intelligence pathological diagnosis should be optimized, and the quality control of the pathological sections included in the artificial intelligence reading should be carried out first to exclude the influence of poor section quality on the computer model. For cases of micrometastasis, we suggest that by differentiating slices into high- and low-confidence groups, low-confidence micrometastatic slices can be separated for manual identification. The normal lymph node structure can be improved by adding samples and training the model in a targeted manner.

**Supplementary Information:**

The online version contains supplementary material available at 10.1186/s13000-023-01432-7.

## Background

According to the 2020 global cancer burden statistics released by the World Health Organization’s International Agency for Research on Cancer [1], the number of new cases of breast cancer in the year reached 2.26 million, replacing lung cancer for the first time and becoming the world’s highest-ranking malignant tumor. Lymph node metastasis is an important factor in the pathological and clinical staging of breast cancer. With the maturity of sentinel lymph node biopsy (SLNB), SLNB has become the standard surgical procedure for preoperative clinical axillary lymph node-negative patients [2]. However, the interpretation of sentinel lymph nodes is cumbersome and time consuming, which greatly increases the workload of pathologists. In 1998, marked by ImageChecker breast computer-aided diagnosis technology system 3 developed by R2 company in the United States [3], artificial intelligence officially entered the field of breast cancer pathological diagnosis. Compared with human interpretation, artificial intelligence interpretation has the advantages of high efficiency, objectivity and repeatability and can analyze a large amount of data instantaneously. Therefore, the field of sentinel lymph node diagnosis, with particular emphasis on rapidity and accuracy, has always been the frontier and hot spot of artificial intelligence research on breast cancer.

The 2016 Camelyon16 Contest [4] was the first major competition in the field of artificial intelligence for sentinel lymph node identification in breast cancer. The best team achieved a reliability of 0.994 at the full-slice level but did not explain the causes of identification errors. The subsequent Camelyon 17 Contest [5] clearly stated that ITC isolated tumor cells, normal lymph node structures (such as small nerves), and contamination during slide preparation were major factors affecting recognition accuracy. Steinbrener et al. [6] proposed an algorithmic solution for identifying false positives due to contamination. The HeLP 2018 Contest [7], published in 2020, used deep learning algorithms to interpret breast cancer lymph node metastasis in frozen sections. The final result reached a confidence level of 0.984 and suggested that neoadjuvant therapy would affect recognition accuracy. However, the existing research has less statistical analysis of the causes of errors, and the proposed solutions are mostly limited to the improvement of algorithms and AI models; no methods are proposed from the perspective of pathology.

Qualitative research is a method focusing on nonquantitative statistics [8]. Complex descriptive data with multiple factors have a unique analytical advantage [9]. The induction of the causes of artificial intelligence identification errors is completed by pathologists. The text information belongs to descriptive data. To make full use of the data information and ensure the credibility of the research, we use a qualitative analysis method to grasp the key word frequency of the text of the causes of errors and deconstruct the causes of errors according to the importance level.

In summary, our study constructed the RRCART deep learning model, used 18 deep learning models to identify 2362 frozen sections of sentinel lymph nodes in breast cancer, obtained the accuracy and analyzed the causes of wrongly identified sections. The reasons for wrongly identified sections were analyzed by constructing an “artificial intelligence RRCART model to identify frozen sections of breast cancer lymph nodes.” Through this work, we hope to screen out the factors that have a greater impact on the accuracy of artificial intelligence sentinel lymph node recognition, propose corresponding improvement directions from a pathological perspective, and clarify the limitations that artificial intelligence currently cannot overcome.

## Methods

### Case selection

We retrospectively collected 2362 frozen sentinel lymph node sections from 499 patients who underwent intraoperative frozen sentinel lymph node biopsy at the Chinese Academy of Medical Sciences Cancer Hospital from January 2017 to December 2019. Participants in the study were randomly sampled. Our cohort included 482 lymph node metastasis sections and 1879 lymph node nonmetastasis sections that were diagnosed during intraoperative frozen sentinel lymph node biopsy. For the cases of suspected metastases intraoperatively, we also collected postoperative paraffin sections and related immunohistochemical information. Frozen and paraffin sections were stained with hematoxylin-eosin, and the slice thickness was 10 μm. We scanned the WSI images of these sections with a Leica Aperio AT2 scanner at a magnification of 40X. The clinicopathological information of the above patients was collected, including sex, age, surgical procedure, final pathological diagnosis, etc.

Our study was approved by the NCC Ethics Committee/IRB (NCC2435). Participants in this study did not have the expected risk, so patient consent was waived.

### Slide reviewing standard

All lymph node sections were first annotated manually by 2 residents with 2–5 years of work experience and then reviewed by senior breast subspecialists (attending doctor and above) with a mean working experience of 14.3 years (9–18 years) in order to better eliminate the bias from individual diagnostic criteria. For suspected metastases, the annotation and reviewing of postoperative paraffin sections and immunohistochemical results were performed by senior breast subspecialists. According to the American Joint Committee on Cancer (AJCC 8th) [10], the type of lymph node metastasis was divided into three groups: micrometastasis (≤2 mm), macrometastasis (> 2 mm) and negative. Annotations were made with the open-source tool ASAP 1.8 (https://github.com/computationalpathologygroup/ASAP/releases). Aiming at the wrongly identified sections per AI, we linked the heatmap identified by the computer to the histological image. The computer engineer and the pathologist jointly determined the specific location of the identification error on the slice and analyze its pathological features from the perspective of histopathology as the reason for the error.

### Software and hardware

The AI software used in this study was the “AI RRCART Model,” which mainly includes three parts. The first is the deep learning module. To confirm the impact of different deep learning models and relevant hyper-parameters on the recognition accuracy of the WSIs, the AI software includes 18 deep learning models, and the recognition results of metastasis can be obtained by every deep learning model. The second is the RandomForest module. Based on the recognition results of metastasis, the AI software uses RandomForest to confirm the metastasis’ 0/1-classification label. The third is the RRCART module. According to the above recognition and classification results, the RRCART model is used to verify whether the WSI is hardly recognized. If the result was in the affirmative, it implied that AI software was unable to correctly recognize the WSI and the WSI needed to be reviewed by pathologists; if not, the implication was that the AI software was working well, and the result just need to be confirmed by pathologists. This work analyzes the causes for the error recognition of WSIs by the “AI RRCART Model,” which requires statistical analysis for the recognition results of metastasis on WSIs. For convenience, the recognition results of metastasis by the “AI RRCART Model” are noted as the recognition results of metastasis by the 18 deep learning models in this work.

The working software environment of the “AI RRCART Model” was Python (version 3.6), OpenSlide (version 1.1.1), TensorFlow (version 1.8.0), scikit-learn (version 0.23), and the hardware environment was an NVIDIA Tesla P40 Graphic Processing Unit (GPU) card.

### Statistical analysis

In this study, we used frequency and error ratio to analyze the importance of each false reason. We defined frequency = the number of cases that were incorrect for one reason/the total number of false cases*100%. The error ratio = the number of models that are incorrect for one reason/the total number of models that are incorrect for all reasons*100%.

Descriptive statistical analysis was performed on the false positive, false negative and corresponding recognition errors of the computer, and further stratified statistical analysis was performed on the high-frequency and low-frequency errors of the slice structure. The research was based on the “Grounded Theory,” and the qualitative research software NVivo 12 was used to input the cause of the error of each incorrectly recognized slice. We encoded the qualitative text of the error cause and formed the parent node and the child node by merging and classifying the free nodes to visualize of the importance of the slice structure and identify the cause of the error (NVivo 12 Plus (Windows12.2.0.443)).

## Results

### Clinicopathological characteristics and error state

There were 498 female patients (99.80%) among the 499 cases. The median age was 51 years (15–84 years). The T stage of 445 patients (89.18%) was less than that of T1a (maximum diameter of infiltration ≤5 mm). The histological type of 83.17% (415 cases) of patients was nonspecific invasive ductal carcinoma (Table 1).

**Table 1:** Basic characteristics of the dataset

Category	Number of patients (%)
**Sex**	
Female	498 (99.80%)
Male	1 (0.20%)
Age (years)^a^	51 (15–84)
**Slide**	
Left	248 (49.70%)
Right	251 (50.30%)
**Tumor Size (mm)**	
≤2	265 (53.11%)
2–5	180 (36.07%)
>5	19 (3.81%)
Missing	35 (7.01%)
**Tumor type**	
IDC	415 (83.17%)
ILC	11 (2.20%)
IBC, other subtypes	35 (7.01%)
DCIS	33 (7.01%)
Missing	5 (1.00%)
Total	499

*IDC* Invasive ductal carcinoma, *DCIS* Ductal carcinoma in situ, *ILC* Invasive lobular carcinoma, *IBC* Invasive breast carcinoma; ^a^ mean (range)

**Fig. 1:**
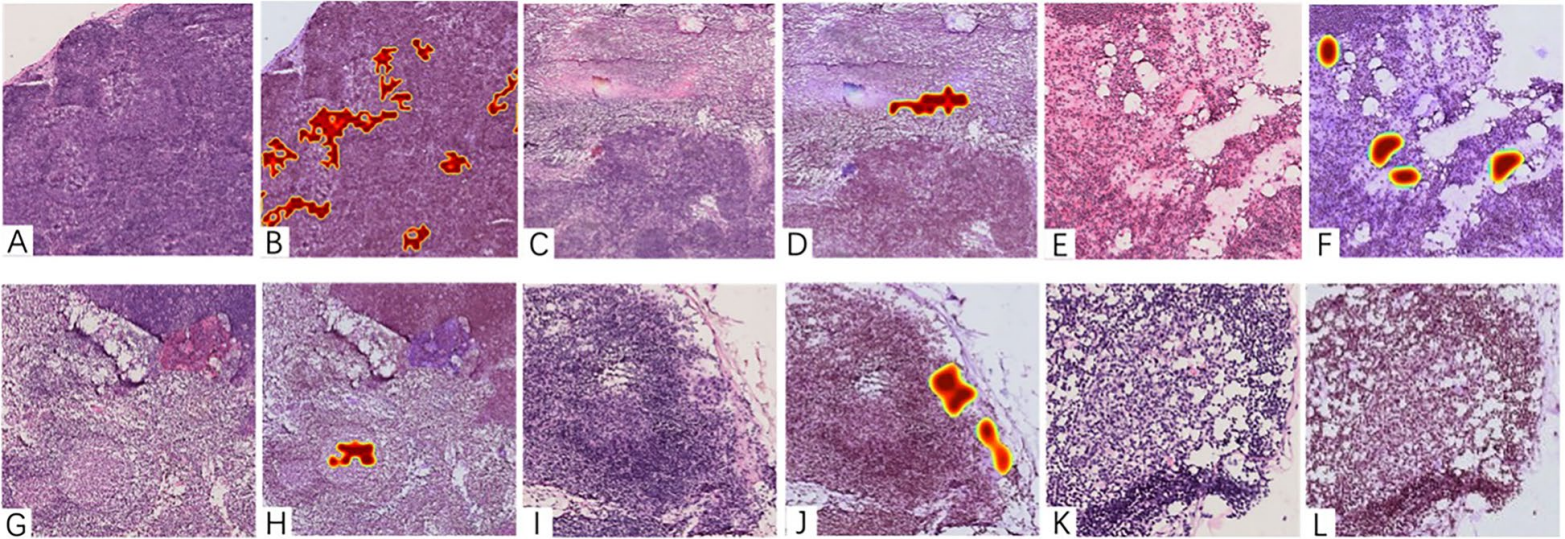
Histological image and heatmap of the wrongly identified section: **A**-**B**: false positive caused by normal structure of lymph nodes (lymphoid follicles); **C**-**D**: false positive caused by low-quality slide (balsam overflow); **E**-**F**: false positive caused by lymph node reactive hyperplasia (lymphedema); **G**-**H**: false positive caused by complex reason (low-quality slide leads to wrong identification of normal structure); **I**-**J**: false negative caused by micrometastasis (model identified but excluded as interference); **K**-**L**: false negative caused by micrometastasis (model unidentified)

Among the 2362 frozen sections collected, there were 101 incorrectly recognized slides in the process of artificial intelligence diagnosis, including 58 false positives (57.43%) and 43 false negatives (42.57%). Through the comparison between the histological image of the wrong slice and the heatmap and subsequent word frequency analysis (see 3.2 for details), we summarized the causes of errors into five categories: low-quality slide, normal lymph node structure, lymph node reactive hyperplasia, micrometastasis, and tumor special growth pattern (Fig. 1). Among them, the cause that occurred at highest frequency was normal lymph node structure with a total of 45 cases (44.55%), followed by micrometastasis (30 cases, 29.70%), slide quality (12 cases, 1.88%), tumor special growth pattern (7 cases, 6.93%), normal lymph node structure identification error caused by poor slide quality (6 cases, 5.94%), and lymph node secondary reaction (1 case, 0.99%). The error ratio for normal lymph node structure was 10.53%, of which the false positive error ratio was 20.59% and the false negative error ratio was 79.41%, indicating that this reason accounted for a higher proportion of false negative results than false positives. The error ratios for other reasons and the respective error ratios for false negatives and false positives are shown in the table (Table 2).

**Table 2:** False-reason divisions

Reason	No.	Frequency (%)	Error ratio (%)	False positive		False negative	
				Frequency (%)	Error ratio (%)	Frequency (%)	Error ratio (%)
Low-quality slide	12	11.88	10.53	58.33	20.59	41.67	79.41
Normal structure	45	44.55	43.50	100.00	100.00	0.00	0.00
Reactive hyperplasia	1	0.99	0.15	100.00	100.00	0.00	0.00
Micrometastasis	30	29.70	33.75	0.00	0.00	100.00	100.00
Special growth pattern	7	6.93	8.98	0.00	0.00	100.00	100.00
Complex reason	6	5.94	3.10	100.00	100.00	0.00	0.00
	101	100.00	100.00	/	/	/	/

Complex reason: Low-quality slide + Normal structure

We define high-frequency errors as occurring in more than 6 of the 18 models and low-frequency errors as occurring in less than 6 models. In 101 incorrectly identified slices, 44 (43.56%) had high-frequency errors, and 57 (56.44%) had low-frequency errors. We believe that low-frequency errors may be more relevant to the model itself, while high-frequency errors indicate that the pathological image itself may have factors that affect the recognition accuracy, making it more meaningful to analyze and improve from the pathological perspective. Among the high-frequency errors, the relatively high error causes included low-quality slides (75%), followed by the identification error of the normal structure of lymph nodes caused by low slide quality (66.67%), indicating that low slide quality factors will not only affect the accuracy as an independent factor but also increase the probability of identification errors of other factors as a confounding factor. It is worth noting that there was only one case of lymph node reactive hyperplasia, and the high-frequency error rate was 100%, indicating that although this reason is rare, once it occurs, it will cause a higher risk of error (Table 3).

**Table 3:** Descriptive statistics of error reasons

Reason	High frequency					Low frequency				
	Frequency (%)	False positive		False negative		Frequency (%)	False positive		False negative	
		Frequency (%)	Error ratio (%)	Frequency (%)	Error ratio (%)		Ratio (%)	Error ratio (%)	Frequency (%)	Error ratio (%)
Low-quality slide	25.00	0.00	0.00	100.00	100.00	75.00	77.78	66.67	22.22	33.33
Normal structure	46.67	100.00	100.00	0.00	0.00	53.33	100.00	100.00	0.00	0.00
Reactive hyperplasia	0.00	0.00	0.00	0.00	0.00	100.00	100.00	100.00	0.00	0.00
Micrometastasis	46.67	0.00	0.00	100.00	100.00	53.33	0.00	0.00	100.00	100.00
Special growth pattern	57.14	0.00	0.00	100.00	100.00	42.86	0.00	0.00	100.00	100.00
Complex reason	33.33	100.00	100.00	0.00	0.00	66.67	100.00	100.00	0.00	0.00

Complex reason: Low-quality slide + Normal structure

### Error reason word frequency analysis and nodal analysis

**Fig. 2:**
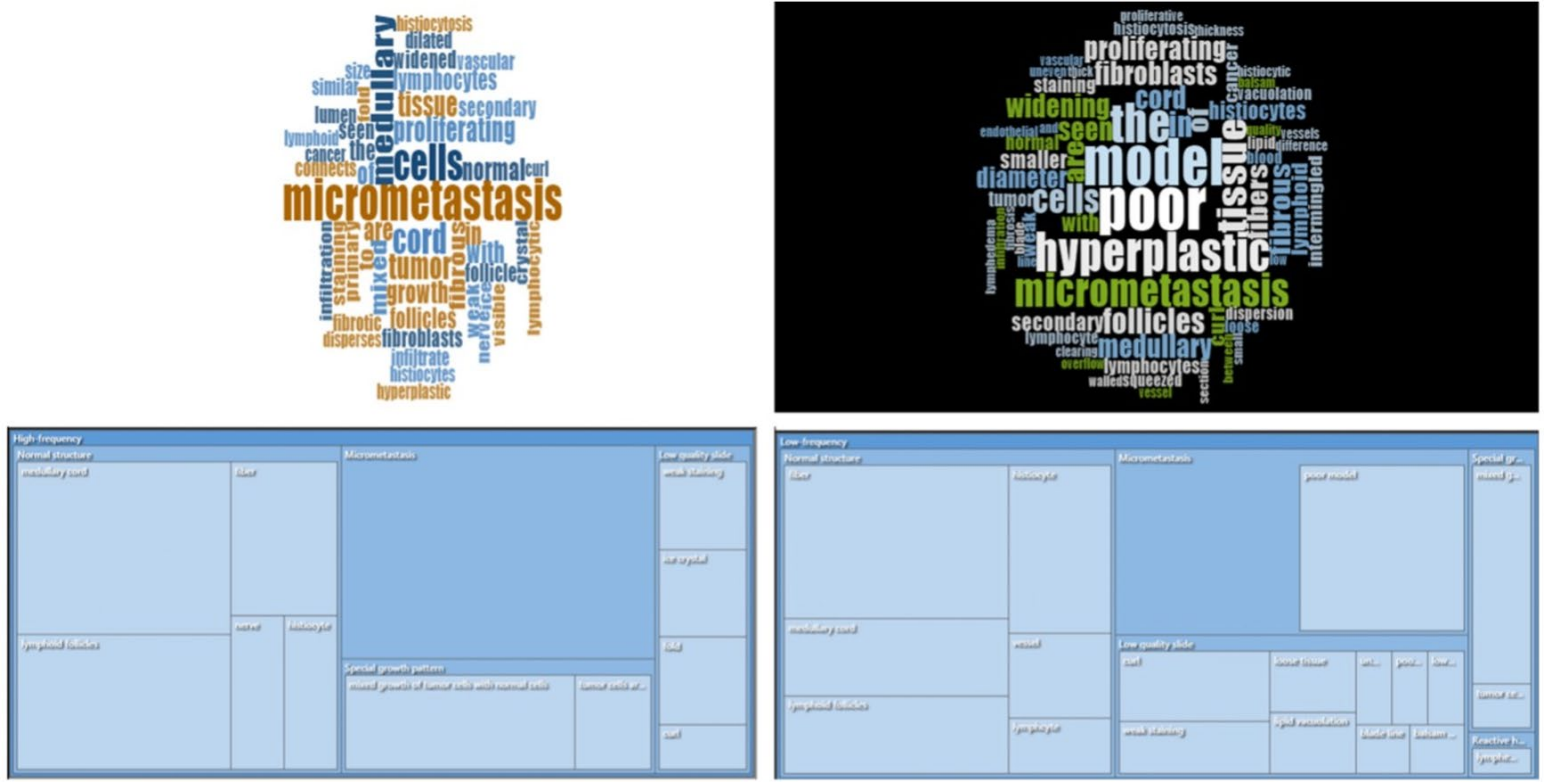
Wordles according to the frequency based on the frequency of occurrence: Left: wordle of high-frequency false reasons; Right: wordle of low-frequency false reasons

Using the word frequency visualization function of NVivo software, we analyzed the causes of errors, counted the frequency of specific words in the text, and drew the wordle according to the frequency of occurrence (Fig. 2). The word frequency area in the figure is positively correlated with the occurrence frequency. As seen from the figure, fibrous tissue, hyperplasia, small diameter, lymphocytes, myelin and other words for high-frequency vocabulary were high frequency factors leading to model identification errors.

We further encoded the nodes of the error reasons. The free nodes are compiled by open coding, and the tree nodes are compiled by spindle coding and selection coding. Finally, two parent nodes (high-frequency error, low-frequency error) and five child nodes (low-quality slide, normal structure, reactive hyperplasia, micrometastasis, special growth pattern) were obtained (Table 4). It can be seen that the number of reference points of normal structure was the largest in both high frequency error and low-frequency error child nodes (medullary cord, lymphoid follicles, fibers, tissue cells, nerve bundles, etc.), indicating that there were more error details in the category of error causes of normal lymph node structure, which will cause computer identification obstacles to varying degrees. In addition, micrometastasis had more reference point values in high-frequency errors.

**Table 4:** Divisions and child nodes of error reasons

Division	First-level child node	Second-level child node^a^	Numbers
High-frequency	Low-quality slide	weak staining, ice crystal, curl, fold	5
	Normal structure	medullary cord, lymphoid follicles, fiber, histiocyte, nerve	25
	Micrometastasis	micrometastasis	12
	Special growth pattern	mixed growth of tumor cells with normal cells, tumor cells are similar in size to lymphocytes	8
Low-frequency	Low-quality slide	Curl, loose tissue, weak staining, lipid vacuolation, ice crystal, low-quality section, blade line, balsam overflow, uneven thickness, poor clearing	19
	Normal structure	Fiber, medullary cord, lymphoid follicles, histiocyte, lymphocyte, vessel	37
	Reactive hyperplasia	lymphedema	1
	Micrometastasis	Poor model, micrometastasis	14
	Special growth pattern	mixed growth of tumor cells with normal cells; tumor cells are similar in size to lymphocytes	6

^a^ In descending order of value

## Discussion

The accuracy of artificial intelligence in identifying sentinel lymph nodes of breast cancer has reached a relatively high level [11]. Based on previous studies, our study used qualitative analysis methods, including word frequency analysis and nodal analysis, to construct the error reason framework of the “artificial intelligence RRCART model to identify frozen sections of breast cancer lymph nodes” by counting the wrong sections and their associated reasons. The results show that the error reasons included two parent nodes (high frequency error, low-frequency error) and 5 child nodes (low-quality slide, normal structure, reactive hyperplasia, micrometastasis, special growth pattern). Among them, the highest frequency was the identification error caused by normal lymph node structure, followed by micrometastasis.

Compared with the results of previous studies [6, 7, 12], our study has the following characteristics. First, our study used intraoperative frozen sections as the research object and included a certain number of cases after neoadjuvant chemotherapy, covering the current inspection of sentinel lymph nodes in the two different clinical conditions. The dataset is representative. Second, we included 18 different deep learning networks through the RRCART model and classified the reasons for errors into high-frequency and low-frequency. We believe that high-frequency errors are more relevant to the slide itself. Third, for the first time, we used the qualitative analysis method to analyze the reasons for errors, which is more scientific and provides a new statistical analysis idea for the literal and nonquantitative data regarding the reasons for errors.

In this study, we deconstructed the reasons for misidentification and innovatively found the complex causes for the impact of low-quality slides on the misidentification of other factors. In other words, in addition to affecting the recognition accuracy as an independent factor, the slide quality problem increases the probability of recognition errors caused by other factors as a confounding variable, making the model unclear about the recognition of normal structures. This shows that the slide quality should actually be the first factor to be recognized, and avoiding low-quality slide error can reduce the composite errors that involve a combination with other factors. Therefore, we believe that slice quality should be considered a prerequisite in the whole process of artificial intelligence training and testing. Before the slide is included in the artificial intelligence model, slice quality control should be carried out first. A high-quality frozen slice should be 1. complete; 2. uniform in thickness; 3. no wrinkles, no folding; 4. no ice crystals, no contamination; 5. good transparency, no overflow of liquid; 6. clear nucleus and pulp; 7. red and blue moderately; 8. clear coloring; 9. correctly placed (Supplement Fig. 1). The slices with obvious curls, weak staining, ice crystals and blade lines (Supplement Fig. 1) are classified in the group of unqualified slices and are not included in the model training.

For the errors caused by normal lymph node structure, we believe that the training of related structures should be added to the training of the model. We found that medullary cord, lymphoid follicles, fibers and other causes of misidentification have the highest incidence. In this regard, we propose the following suggestions from two perspectives. First, referring to the relevant literature [13], we suggest that the normal lymph node structure that is misidentified in the section can be annotated and reentered in the model for training to remove false positives. Second, we collected and annotated normal structures, such as the medullary cord, lymphoid follicles, and fibers, and trained the model for normal structure identification. This will also be the direction of our next study.

For the errors caused by micrometastasis, based on the relevant data [14], we believe that when the metastasis is too small, the current manual labeling method limits the judgment of the computer model so that it cannot fully recognize all the diameters of the micrometastases. In the process of labeling and model training, we also found that the minimum threshold for model recognition of metastases was related to the labeler. After the senior pathologist reviews the labeled model, the recognition ability of micrometastasis will be improved. In addition, we found that the maximum diameter of all the micrometastases identified as false negatives in the RRCART model was 0.9141 mm, less than the 2 mm required for N1a staging. Existing guidelines and related studies have shown that nonradical surgical treatment in the presence of SLN micrometastasis in breast cancer patients does not lead to local recurrence and distant metastasis [10, 15]. Therefore, we recommend that micrometastasis sections with low confidence be manually identified by differentiating slide confidence. A related methodological article has been published [16].

There are still some limitations in our research. First, our dataset is from a single center, and the consistency, robustness and frequency of error reasons of the model are not verified with a large sample and multicenter sections. However, our dataset covers different clinical conditions and has a certain representativeness. We will launch multicenter research verification in the next step and increase the sample size. Second, we only summarize the reasons for the errors and propose a preliminary solution. Next, we will further explore the solutions.

## Conclusions

The reasons for errors in the identification of sentinel lymph node frozen sections by artificial intelligence from high to low were normal structure, micrometastasis, low-quality slide, special tumor growth pattern and reactive hyperplasia. In addition, we found that the presence of low-quality slides can be combined with other factors as a confounding factor to increase the probability of false identification. Therefore, we suggest that the process of artificial intelligence pathological diagnosis should be optimized, and the quality control of pathological sections included in artificial intelligence reading should be carried out first to eliminate the influence of low-quality sections on the computer model. For cases of micrometastasis, we believe that AI cannot accurately identify metastases that are too small and require manual diagnosis. As an error reason, normal structure can be addressed by an increased amount of sample and targeted training of the model.

### Supplementary Information

**Additional file 1: Supplement Figure 1.** Comparison of good quality frozen section slide and poor quality frozen section slide. A: good quality frozen section slide; B: poor quality frozen section slide.

## Data Availability

The data are not publicly available due to hospital regulations. But data requests with aims will be needed to assess the reasonability. After approval from the hospital and the corresponding authors, de-identified clinical data will be provided.

## Abbreviations

SLNB: Sentinel lymph node biopsy
AI: Artificial intelligence
RRCART: Relative risk classification regression tree
WSI: Whole-slide image
NCC: National Cancer Center
AJCC: American joint Committee on cancer
SLN: Sentinel lymph node
