# Supplementary material for: Analysis of false reasons based on the artificial intelligence RRCART model to identify frozen sections of lymph nodes in breast cancer
Source: Diagn Pathol. 2024 Jan 22;19:18. doi: 10.1186/s13000-023-01432-7 (PMC10802064; doi:10.1186/s13000-023-01432-7)
Supplement: Supplementary file 1 — Additional file 1: Supplement Figure 1. Comparison of good quality frozen section slide and poor quality frozen section slide. A: good quality frozen section slide; B: poor quality frozen section slide. [file 13000_2023_1432_MOESM1_ESM.docx]

**
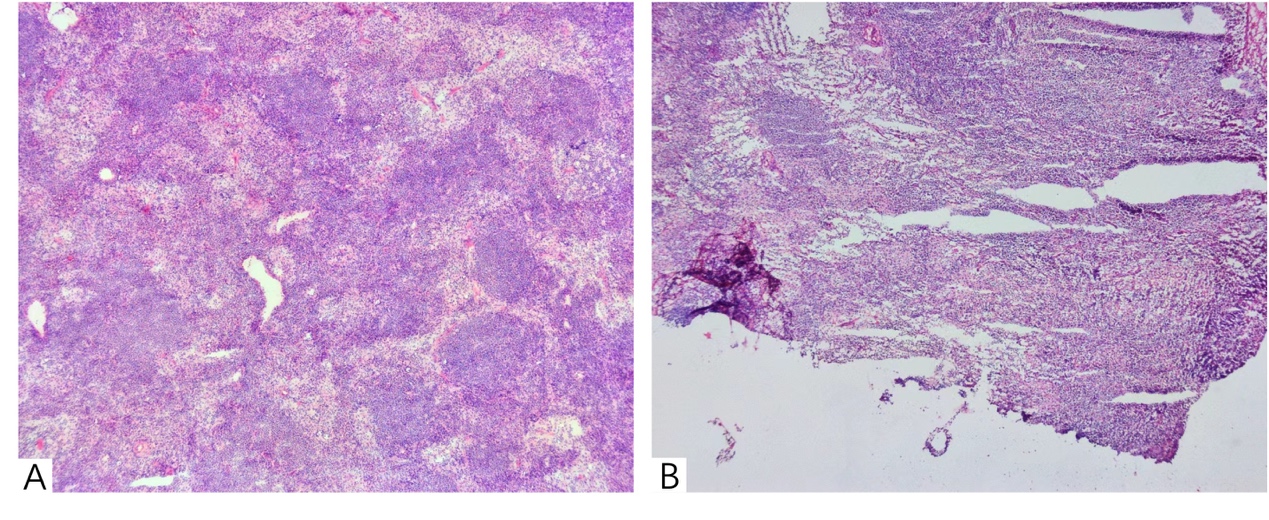
**

**Supplement Figure 1:** Comparison of good quality frozen section slide and poor quality frozen section slide. A: good quality frozen section slide; B: poor quality frozen section slide.
